# Expression Profile Analysis Identifies a Novel Seven Immune-Related Gene Signature to Improve Prognosis Prediction of Glioblastoma

**DOI:** 10.3389/fgene.2021.638458

**Published:** 2021-02-23

**Authors:** Li Hu, Zhibin Han, Xingbo Cheng, Sida Wang, Yumeng Feng, Zhiguo Lin

**Affiliations:** Department of Neurosurgery, The First Affiliated Hospital of Harbin Medical University, Harbin, China

**Keywords:** glioblastoma, expression profile, immune-related genes, prognosis prediction, overall survival

## Abstract

Glioblastoma multiform (GBM) is a malignant central nervous system cancer with dismal prognosis despite conventional therapies. Scientists have great interest in using immunotherapy for treating GBM because it has shown remarkable potential in many solid tumors, including melanoma, non-small cell lung cancer, and renal cell carcinoma. The gene expression patterns, clinical data of GBM individuals from the Cancer Genome Atlas database (TCGA), and immune-related genes (IRGs) from ImmPort were used to identify differentially expressed IRGs through the Wilcoxon rank-sum test. The association between each IRG and overall survival (OS) of patients was investigated by the univariate Cox regression analysis. LASSO Cox regression assessment was conducted to explore the prognostic potential of the IRGs of GBM and construct a risk score formula. A Kaplan–Meier curve was created to estimate the prognostic role of IRGs. The efficiency of the model was examined according to the area under the receiver operating characteristic (ROC) curve. The TCGA internal dataset and two GEO external datasets were used for model verification. We evaluated IRG expression in GBM and generated a risk model to estimate the prognosis of GBM individuals with seven optimal prognostic expressed IRGs. A landscape of 22 types of tumor-infiltrating immune cells (TIICs) in glioblastoma was identified, and we investigated the link between the seven IRGs and the immune checkpoints. Furthermore, there was a correlation between the IRGs and the infiltration level in GBM. Our data suggested that the seven IRGs identified in this study are not only significant prognostic predictors in GBM patients but can also be utilized to investigate the developmental mechanisms of GBM and in the design of personalized treatments for them.

## Introduction

Glioblastoma constitutes the most recurrent and aggressive primary malignant tumor of the central nervous system (Yan et al., 2012). In spite of multimodal conventional treatments consisting of neurosurgical resection as well as radiotherapy with accompanying adjuvant alkylating agent temozolomide chemotherapy, the prognosis for glioblastoma multiform (GBM) individuals remains dismal, with a median survival time ranging from 9.4 to 19.0 months (Yang et al., 2014). This poor outcome is due to the highly invasive nature, malignant progression, drug resistance, and tumor recurrence, which are regulated by a large number of oncogenes and tumor suppressor genes (Liu et al., 2015; Cao et al., 2019). Next-generation sequencing technologies have made great progress recently, enabling scientists to gain profound insights into the molecular level of GBM pathophysiology (Aldape et al., 2015). As a consequence, many prospective diagnostic and prognostic biosignatures have been discovered, which enable a more distinct classification and a more precise outcome estimation of GBM. Nonetheless, given the dismal prognosis of GBM, a multiple-gene signature derived model is still urgently required to estimate the prognosis and treatment response more accurately for GBM patients.

The immune microenvironment has been chronicled to play a pivotal function in tumor biology (Hanahan and Weinberg, 2011), and cancer immunotherapy has been demonstrated to have a significant preclinical or clinical value to many patients with some sensitive types of cancer (Schumacher and Schreiber, 2015; Steven et al., 2016; Odunsi, 2017; Morrison et al., 2018; Christofi et al., 2019). Increasing research evidence supports the idea that although the brain constitutes an immunologically specific site, the immune microenvironment provides ample opportunities for immunotherapy of brain tumors (Lim et al., 2018). Many kinds of immunotherapy, including GBM vaccines, oncolytic viral therapies, immune-checkpoint suppressors, and chimeric antigen receptor T cell therapy, have been tested in clinical trials, but the results are not satisfactory. Tumors are insensitive to immunotherapy due to the immunosuppressive tumor microenvironment, defects in tumor antigen presentation, and characteristics of the physical microenvironment, including hypoxia and necrosis (Lim et al., 2018; Pombo Antunes et al., 2020). The precise mechanism of immune escape is unclear. Glioblastoma usually has a low mutational load and lower T cell invasion relative to other tumor types (Li et al., 2016). Thus, it is imperative to better comprehend the progress and mechanisms of the GBM immune microenvironment. Multiple recent studies have suggested that immune gene expression profile biosignatures may be used as a prediction for clinical outcomes in many cancers (Bremnes et al., 2016; Campbell et al., 2017; Öjlert et al., 2019). Li et al. (2017) created a personalized immune-related gene prognostic biosignature to improve the prognosis of individuals with NSCLC.

In a previous study, a prognostic immune-related gene signature with nine IRGs based on a total of 161 samples from the Cancer Genome Atlas database (TCGA) was generated (Liang et al., 2020), and the 9-IRG model was identified as an independent predictor in glioblastoma. These researchers established a crosstalk network between prognostic immune-related genes (IRGs) and transcription factors. Correlations between immune infiltration cells and risk score were also identified. However, the potential molecular mechanisms were not clarified in their study. Thus, it is necessary to elucidate the function of these genes in the risk score and poor survival outcomes.

Here, we generated a seven immune-linked gene biosignature to exhibit the connection between gene expression and GBM prognosis, and we verified this biosignature in the TCGA and GEO dataset. These data may provide a novel reference for the prognostic prediction of GBM. We also confirmed the relevance of the seven IRGs to immune checkpoints, immune cell infiltration, oncogenesis pathway, and drug sensitivity. As a result, we not only generated a predictive model for GBM prognosis but also indicated the potential function of these IRGs in the occurrence and development of glioblastoma.

## Materials and Methods

### Data Sources and Preliminary Processing

The RNA-Seq data of 169 GBM samples and five normal brain samples, as well as the clinical data of these GBM patients, such as age, gender, molecular subtype, gene mutation status, survival time, and survival status, were obtained from the TCGA dataset^1^. Additionally, the GBM patients’ microarray and clinical data were collected from independent datasets in the GEO database, including GSE74187 (*n* = 60) and GSE4412 (*n* = 59). These gene expression data were generated and annotated on GPL6480 or GPL97 platform. The immune-related gene set, including 2,498 genes, was downloaded from the ImmPort database. The RNA-Seq and microarray data were normalized using scale method, and the data were pre-processed through the following steps: (1) patients with unavailable clinical and/or survival information were removed, (2) only the expression profiles of IRGs were preserved, and (3) genes with exceeding low abundance were filtered out (the expression value was 0 in more than half of the samples, or the average expression value was less than 0.3 in the samples). Finally, 1,100 genes were used for univariate Cox regression analysis and LASSO analysis.

### Differential Gene and Functional Enrichment Analysis

The expression analysis of 2,498 immune-linked genes was conducted to identify the differentially expressed IRGs by the limma R package [false discovery rate (FDR) < 0.05 and log_2_ | fold change| > 1] (Ritchie et al., 2015). We conducted functional enrichment analyses to identify potential molecular biomechanisms of the differentially expressed IRGs *via* GO analysis and KEGG pathways (Yang et al., 2018). GOplot package was used for illustrating the relationship between genes and enriched KEGG pathways. Gene Set Enrichment Analysis (GSEA) (Mootha et al., 2003; Subramanian et al., 2005) was employed to examine the signaling cascades in which the IRGs were enriched between the high- and low-risk subgroups.

### Establishment of the Immune-Associated Gene Biosignature

The univariate Cox regression analysis was applied to investigate the association between each IRG and OS of patients based on the TCGA dataset. To build the immune-related risk model, the genes with *p* value < 0.01 were considered as candidate survival-associated IRGs. The LASSO regression model was used to determine the most significant survival-correlated IRGs. First, the GBM patients in TCGA dataset were randomly divided into training and internal validation cohorts at a 4:1 ratio, forming a training cohort (*n* = 134) and an internal validation cohort (*n* = 33). The LASSO regression was employed based on 10-fold cross-validation to minimize the risk of overfitting. LASSO tends to “shrink” the regression coefficients to zero as λ increases. The optimal λ that yielded minimum cross validation error in 10-fold cross validation was chosen. The risk score was calculated by using the sum of normalized expression weighted by the LASSO regression coefficients (Zhong et al., 2020):

Risk score=EmRNA1×CmRNA1+EmRNA2×CmRNA2+EmRNAn×CmRNAn

where E designates the expression level of each gene; and C designates the lasso regression coefficient of each gene.

The patients were separated into low- and high-risk groups according to the median of the risk score. OS of the patients in the two groups was analyzed by the log-rank test with “survival” package in R. Receiver operating characteristic (ROC) curve and the corresponding area under the ROC curve (AUC) were calculated to evaluate the prognostic value of the risk score by using “ROC” package.

### CIBERSORT and Assessment of Tumor-Infiltrating Immune Cells

CIBERSORT is a computational technique that predicts the cell type signature in mix tissues through gene expression levels (Newman et al., 2015). Cell types can be identified using RNA mixtures in nearly any tissue (Yang et al., 2019). For this study, we employed CIBERSORT to examine the 22 types of immune cells in tumor tissues and show the percentages of 22 sets of tumor-infiltrating immune cells (TIICs) with bar plots and a corheatmap.

### Analysis of Immune Infiltration

To analyze the correlation between the risk signature and infiltrating levels of six immune cells, including B cells, CD4+ T cells, CD8+ T cells, neutrophils, macrophages, and dendritic cells, Spearman’s correlation was calculated and the strength of correlation for the absolute value of *r* was as follows: *r* between 0 and 0.3 indicates a weak correlation; *r* between 0.3 and 0.7 indicates a moderate correlation; *r* between 0.7 and 1.0 indicates a strong correlation (Akoglu, 2018).

### Statistical Analysis

Boxplot was generated using the “ggplot2” package in R. Heat map was generated using the “pheatmap” package in R. A correlation analysis of the seven immune genes was performed using the R “corrplot” package in the Pearson’s method. Circular plot was generated using the “circlize” package in R. Student’s *t* test was used to compare data from subgroups. Pearson’s correlation test was used to analyze the correlation between the IRGs signature and the expression of immune checkpoint genes. K-M survival curves were compared using log-rank test. All statistical analyses were conducted on R software (version 3.6.0). A *p* value of < 0.05 was considered to indicate significance. Other statistical methods were described throughout the study.

## Results

### Identification of Differentially Expressed IRGs in GBM

**FIGURE 1:**
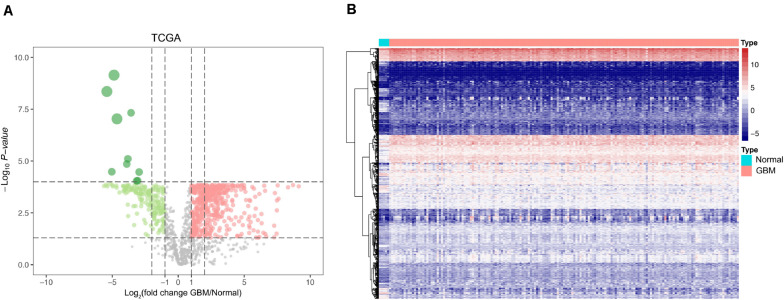
Identification of differentially expressed IRGs between GBM and normal brain tissues. **(A)** Volcano plots showing the log_2_ (fold change) of mRNA in GBM compared to normal brain tissues, and the corresponding-log_10_ (*P* value) in TCGA datasets. Genes with adjusted P value below 0.05 and fold change above one (below –1) were marked with red (green) dots. **(B)** Heatmap of the differentially expressed IRGs in TCGA datasets.

The mRNA levels of 2,498 IRGs in GBM (*n* = 169) and normal brain tissues (*n* = 5) from TCGA were compared *via* the Wilcoxon rank-sum test. In total, 595 differentially expressed IRGs comprising 416 upregulated genes and 179 downregulated genes were identified (Supplementary Table 1). The volcano plot and heat map of differentially expressed IRGs are shown in Figures 1A,B.

### Functional Characterization of DEIRGs

**FIGURE 2:**
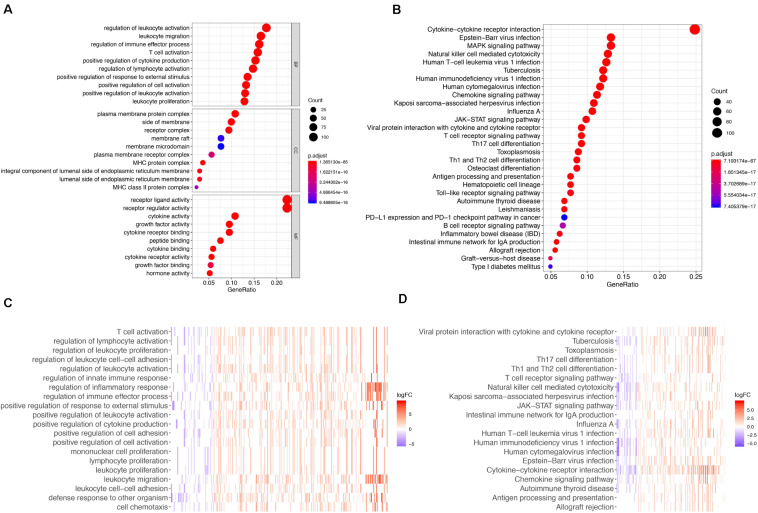
GO terms and Enrichment of KEGG pathways for differentially expressed IRGs. **(A)** GO biological process analysis for the immune-related DEGs. **(B)** KEGG pathway enrichment analysis for the immune-related DEGs. **(C)** Heatmap of the GO terms by logFC. **(D)** Heatmap of the KEGG pathways by logFC.

The gene functional enrichment assessment showed that immune responses were the most common. The most significant biological terms were “regulation of leukocyte activation,” “plasma membrane protein complex” and “receptor ligand activity” among biological processes, cellular components, and molecular functions, respectively (Figure 2A). With regard to the KEGG cascades, most of signaling cascades were linked to immune reactions, and cytokine-cytokine receptor crosstalk was the most significantly enriched term (Figure 2B). For better visualization, two heatmaps of these values were plotted using the logFC, including one for GO terms (Figure 2C) and the other for KEGG pathways (Figure 2D). Some GO terms and KEGG cascades were linked to certain immune processes.

### Identification of Prognostic Genes

**FIGURE 3:**
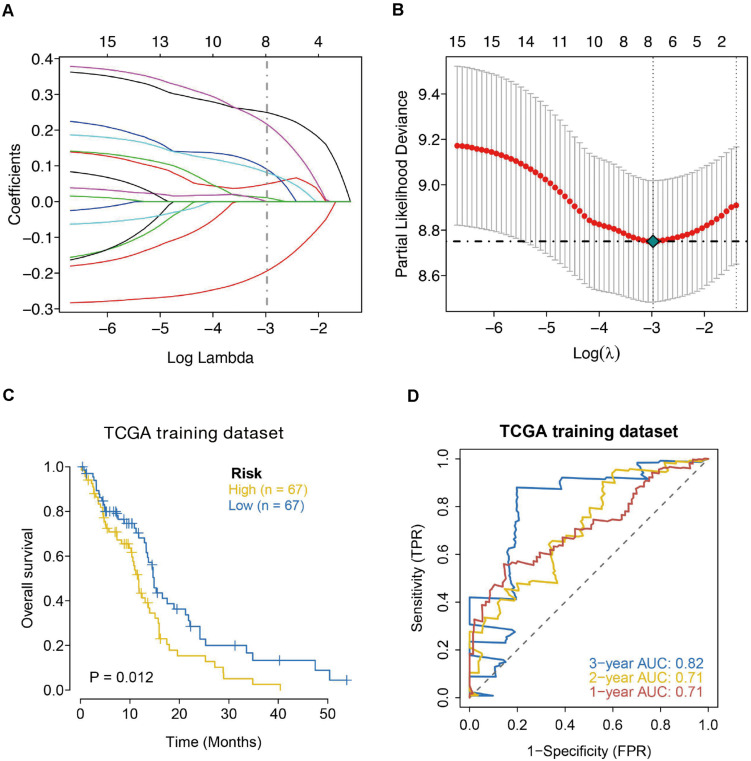
Seven-immune-related gene signature prognostic risk model analysis of GBM patients in TCGA dataset. **(A)** LASSO coefficient profiles of the 15 IRGs in TCGA-GBM. **(B)** A coefficient profile plot was generated against the log (lambda) sequence. Selection of the optimal parameter (lambda) in the LASSO model for TCGA. **(C)** Kaplan–Meier survival curves for high-risk and low-risk groups. **(D)** ROC curves to examine the predictive accuracy of the model for OS at 1-, 2-, and 3- years.

The univariate Cox regression model was applied to select IRGs with the patient OS, and a total of 15 IRGs were discovered to be significantly associated with OS (*p* < 0.01). These genes were subjected to the LASSO regression analysis to calculate the correlation coefficients. The signature performed best when only seven genes were included (Figures 3A,B). For this analysis, we used LASSO regression to obtain the following seven optimal IRGs (risk genes) for incorporation into the prognostic risk model in TCGA training cohort (Supplementary Figure 1): Bone Morphogenetic Protein Receptor Type 1A (BMPR1A), Cathepsin B (CTSB), NFKB Inhibitor Zeta (NFKBIZ), TNF Superfamily Member 14 (TNFSF14), C-X-C Motif Chemokine Ligand 2 (CXCL2), Semaphorin-4F (SEMA4F), and Oncostatin M Receptor (OSMR). Among these genes, CTSB, NFKBIZ, TNFSF14, CXCL2, SEMA4F, and OSMR were characterized as high-risk genes (estimating a poor prognosis), whereas BMPR1A was identified as low-risk genes (functioning as a protective factor) with regard to the OS of patients (see detailed information in Table 1).

**TABLE 1:** Risk genes in the prognostic risk model.

Gene	Coef	HR	Low. 95%CI	Upp. 95%CI	*p*-value
BMPR1A	−0.194	0.691	0.556	0.859	8.86E-4
CTSB	0.011	1.280	1.104	1.484	1.06E-3
NFKBIZ	0.050	1.442	1.200	1.731	9.10E-5
TNFSF14	0.081	1.320	1.138	1.532	2.58E-4
CXCL2	0.090	1.350	1.152	1.583	2.11E-4
SEMA4F	0.217	1.490	1.199	1.852	3.25E-3
OSMR	0.250	1.475	1.239	1.757	1.30E-5

*7 prognostic immune-related genes screened out by the univariate Cox regression and LASSO Cox proportional hazards regression.*

### Construction of a Seven-Gene Prognostic Biosignature

The LASSO regression analysis was used to screen the risk genes for estimating the prognosis of GBM individuals (Friedman et al., 2010; Simon et al., 2011). We utilized mRNA contents and predicted the regression coefficients of the risk genes to compute a risk score for each GBM individual. The prognostic estimation model was created, which incorporated seven immune-linked genes. The following formula was used for the calculation:

Risk⁢score=(-0.194)⁢BMPR1A+0.011⁢CTSB+0.050⁢NFKBIZ+0.081⁢TNFSF14+ 0.090⁢CXCL2+0.217⁢SEMA4F+0.250⁢OSMR

According to the formula, we calculated the risk scores of each GBM individual and clustered them into low-risk and high-risk classes according to the median risk score. According to the log-rank test, the Kaplan–Meier curve revealed that the prognosis in the high-risk class was worse compared to the low-risk class in TCGA training cohort (*p* = 0.012) (Figure 3C). We employed the time-dependent ROC curves to explore the estimation accuracy of the model for OS in TCGA training cohort. The prognostic model area under the ROC values were 0.71 at 1-year, 0.71 at 2-year, and 0.82 at 3-year (Figure 3D). Suggesting our 7-gene model had a favorable efficiency in predicting prognosis.

### Verification of the Immune-Linked Gene Biosignature

**FIGURE 4:**
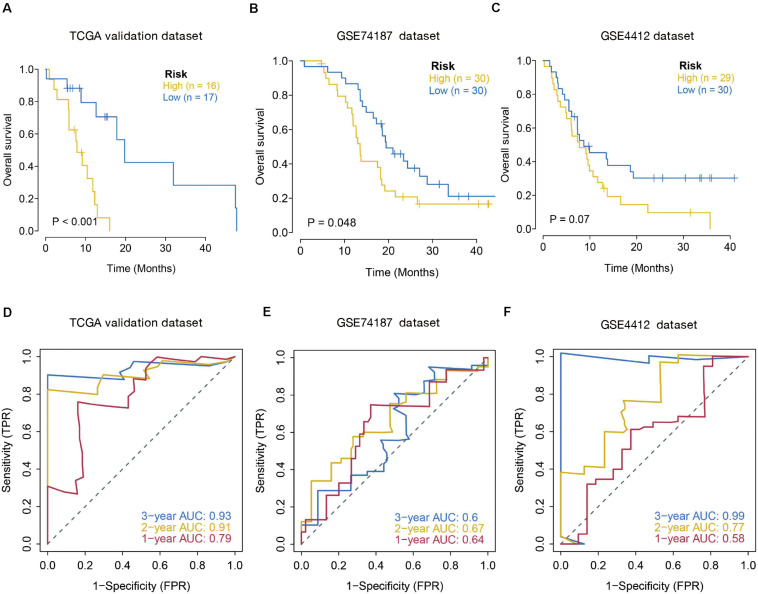
Validation of seven-immune-related gene signature prognostic risk model of GBM patients in validation datasets. (A) Kaplan–Meier survival curves for high-risk and low-risk groups in TCGA internal validation dataset (*p* < 0.001). **(B)** Kaplan–Meier survival curves for high-risk and low-risk groups in GSE74187 dataset (*p* = 0.048). **(C)** Kaplan–Meier survival curves for high-risk and low-risk groups in GSE4412 dataset (*p* = 0.07). **(D–F)** ROC curves to examine the predictive accuracy of the model for OS at 1-, 2-, and 3- years in validation cohorts.

The prognostic value of the seven IRGs signature was further evaluated in three validation sets (TCGA internal validation set, GSE74187, and GSE4412 datasets). The risk score for each patient was calculated following the same formula. Patients in three validation sets were classified into high- and low-risk groups based on the median of the risk score. Survival analysis in the three validation sets confirmed a lower survival rate in the high-risk group (Figures 4A–C). The AUC of ROC curves for 1-, 2-, and 3-year survival rate in the validation dataset were 0.79, 0.91, and 0.93 (TCGA internal validation cohort) 0.64, 0.67, and 0.6 (GSE74187); 0.58, 0.77, and 0.99 (GSE4412) (Figures 4D–F). In summary, the prognosis model created according to the expression patterns of these seven prognosis-distinct immune-linked genes had high estimation accuracy and stability in identifying immune features. These data demonstrated that our prognostic risk model precisely estimates the prognosis of GBM individuals.

### Relationship Between the Risk Score and Clinical Factors

The relationship between the seven IRGs signature and clinical factors, including age, gender, IDH1 mutation, 1p/19q mutation, and subtype was further investigated using data from the TCGA dataset. The results showed that a higher risk score was always associated with IDH1 mutation, 19q mutation, and subtype. No differences were observed between the risk score and age, gender, or 1p mutation (Supplementary Figure 2).

### Functional Annotations and Signaling Pathway Enrichment in High- and Low-Risk Score Groups

**FIGURE 5:**
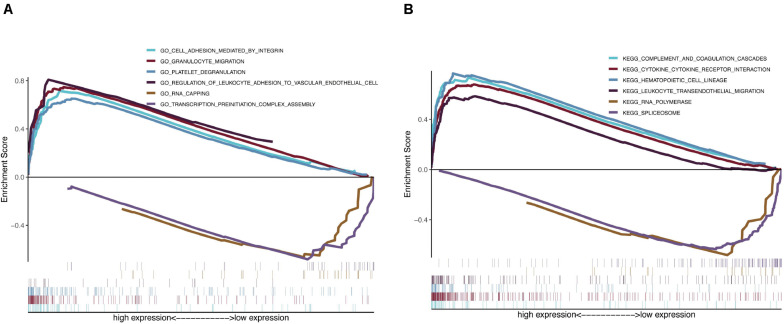
Functional gene annotations and KEGG enrichment analysis between high and low risk groups. **(A)** ClusterProfiler was selected for functional gene annotations. **(B)** GSEA analysis was performed to identify the potential pathways differentiate the high and low risk groups.

Because the monitoring of disease outcome is imperative for clinical management, we aimed to identify molecular biosignatures that could be utilized as viable prognostic indicators. Functional gene annotation and KEGG enrichment analyses focused on the above mentioned seven prognosis-distinct immune-linked genes were conducted (Yu et al., 2012). We demonstrated that these survival-linked IRGs were most abundant in gene ontology (GO) terms linked to “cell adhesion mediated by integrin,” “granulocyte migration,” “platelet degranulation,” “regulation of leukocyte adhesion to vascular endothelial cell,” “rna capping” and “transcription preinitiation complex assembly” (Figure 5A). Gene set enrichment analysis (GSEA) was performed to identify the prospective cascades that differentiated the high- or low-risk groups. The following cascades were significantly enriched: “complement and coagulation cascades,” “cytokine cytokine receptor interaction,” “hematopoietic cell lineage,” “leukocyte transendothelial migration,” “rna polymerase,” and “spliceosome” (Figure 5B). These results suggested that the prognosis-specific immune-related gene risk score using the seven IRGs may affect these cascades and estimate the survival of GBM patients.

### Correlation Between the Risk Score and Immune Response

**FIGURE 6:**
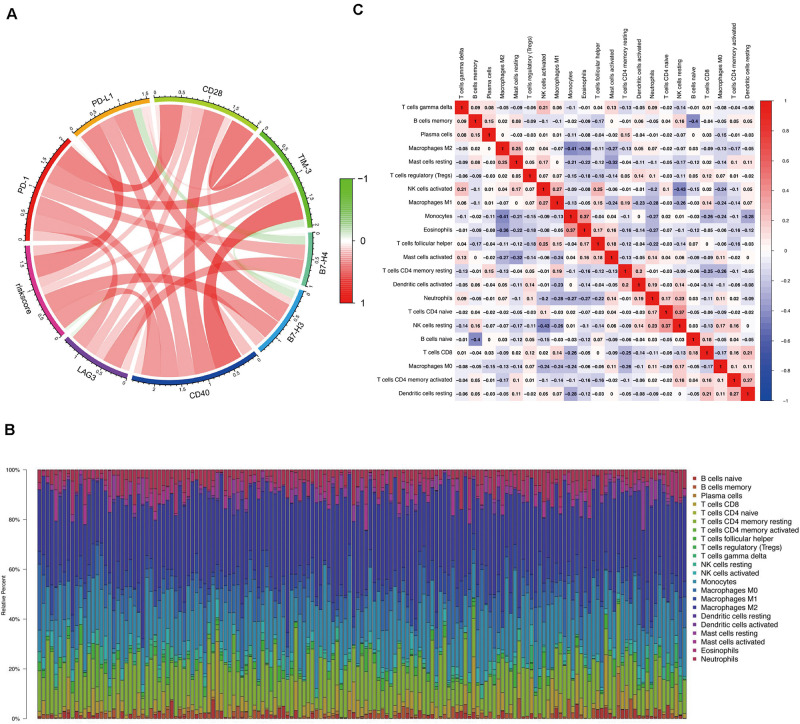
Correlation between the risk score and immune response and the distribution of immune infiltration in GBM. **(A)** Circos plot shows the relationship between the risk score and the expression levels of some important immune checkpoints in GBM. **(B)** The proportions of immune cells in each GBM sample are indicated with different colors, and the lengths of the bars in the bar chart indicate the levels of the immune cell populations. **(C)** Correlation matrix for all 22 immune cell proportions. Some immune cells were negatively related, represented in blue, and others were positively related, represented in red. The darker the color, the higher the correlation.

To better comprehend the connection between the risk score and immune response, we calculated the association between the risk score and the expression levels of core immune checkpoints in GBM, such as CD28, TIM-3, B7-H3, PD-1, B7-H4, CD40, LAG3, and PD-L1. Interestingly, the Circos plot (Gu et al., 2014) showed that the risk score was strongly linked to expression levels of B7-H3, CD40, and PD-L1 in TCGA cohorts (Figure 6A).

### Distribution of Immune Invasion in Glioblastoma

We first assessed immune invasion in glioma tissue in 22 subpopulations of immune cells by employing the CIBERSORT algorithm. In Figure 6B, the percentage of immune cells in each GBM sample is shown in different colors, and the lengths of the bars indicate the immune cell population levels. We then speculated that the divergence in TIIC proportions may function as a critical feature of individual differences and possess prognostic significance. Based on the chart, we established that glioma tissues had comparatively high proportions of M1, M0, and M2 macrophages as well as monocytes, which were responsible for approximately 70% of the 22 subpopulations of immune cells. In contrast, B cell and neutrophil proportions were comparatively low, and they were responsible for approximately 10% of the immune cell subpopulations (Figure 6B). Proportions of different types of immune cells subsets were weakly and then moderately correlated (Figure 6C). Populations with a negative correlation consisted of monocytes/M2 macrophages (Pearson’s correlation = −0.41) and resting NK cells/activated NK cells (Pearson’s correlation = −0.43). Given the important role of these hub immune genes, the genetic variations of five of them with a mutation rate ≥ 5% were further explored (Supplementary Figure 3).

### Prognostic Model Associates With Immune Invasion in GBM

**FIGURE 7:**
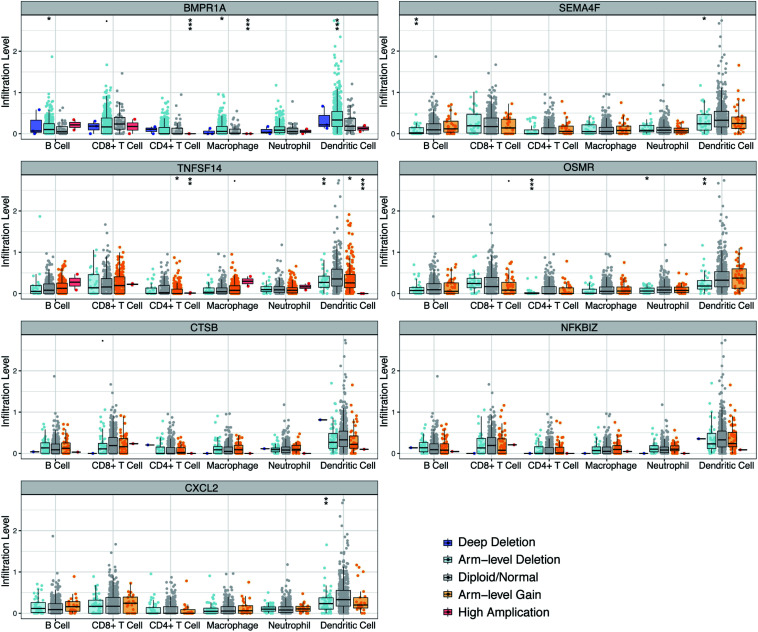
Correlations of seven immune-related gene copy member with immune infiltration level in GBM. These seven-immune-related gene CNV affects the infiltrating levels of different immune cells in GBM. *p < 0.05, **p < 0.01, ***p < 0.001.

Clinical studies on immunotherapy have verified that tumor-invading lymphocytes in the tumor microenvironment possess an estimation significance for prognosis and treatment using immunotherapy in some solid tumors (Bremnes et al., 2016; Lee et al., 2016; Badalamenti et al., 2019). Given that our risk score was centered on seven immune-linked genes, we investigated whether it was linked to the invading levels of six immune cell types in the TCGA GBM cohort acquired from TIMER. We examined the link between the expression levels of seven immune-linked genes and the invading contents of six immune cell types. The findings demonstrated that the expression of these seven genes exhibited remarkably positive correlation with immune cell invasion. The expressions of CTSB, NFKBIZ, CXCL2, and OSMR were all correlated with the invading levels of dendritic cells (Supplementary Figure 4). To better understand the impact of the seven IRGs signature on the infiltration of immune cells, the relevance of the risk score and six immune cells was investigated. Results indicated that the risk score was positively related to neutrophil cells (*r* = 0.188), dendritic cells (*r* = 0.404), and CD4+ T cells (*r* = 0.169) (Supplementary Figure 5). Collectively, these data indicated that our model system is partially linked to the invading level of immune cells in the tumor microenvironment of GBM. Particularly, BMPR1A was significantly correlated with the infiltrating levels of CD4+ T cells, macrophages, and dendritic cells. TNFSF14 and OSMR were significantly correlated with the invading levels of CD4+ T cells and dendritic cells. CXCL2 was significantly associated with the invading levels of dendritic cells (Figure 7).

### Effects of Prognosis-Specific Immune-Related Genes on Oncogenic Pathways

To further elucidate the molecular mechanisms for prognosis-specific IRGs participating in tumorigenesis, we explored the link between the expression of individual genes and activation or repression of 10 core signaling cascades based on a pathway score computed from the sum of the relative protein contents for all positive modulatory constituents less that of all negative modulatory constituents (Akbani et al., 2014). Our data demonstrated that seven genes were highly correlated to the activation or suppression of numerous oncogenic cascades (Supplementary Figure 6). For example, CTSB was highly correlated with the repression of DNA damage response and AR hormone, as well as the activation of apoptosis and EMT signaling pathways. CXCL2 was associated with the inhibition of cell cycle, DNA damage response, and AR hormone, as well as activation of apoptosis, EMT, and RAS/MAPK signaling pathways. These results suggested that prognosis-specific IRGs are linked to alterations of diverse oncogenic cascades.

### Hub Gene Drug Sensitivity

GSCALite constitutes a web-based analysis portal for gene set cancer analysis (Liu C. J. et al., 2018), based on which the drug sensitivity of the hub genes was analyzed to provide support on drug-targeted therapy (Supplementary Figure 7). Low NFKBIZ level is resistant to 11 drugs or small molecules, low BMPR1A level is resistant to seven drugs or small molecules, low SEMA4F level is resistant to 16 drugs or small molecules, and low levels of OSMR, CXCL2, and CTSB are resistant to more than 32 drugs or small molecules.

## Discussion

Glioblastoma is a fatal human cancer. Despite of the years of research focused on GBM biology and the numerous clinical trials to evaluate new treatments, the prognosis of individuals with glioblastoma remains dismal (Thakkar et al., 2014). Patients diagnosed with GBM undergo treatments, including neurosurgery, radiotherapy, and chemotherapy, with unsatisfactory survival.

There has been great advancement in the comprehension of the genetic and molecular underpinnings of glioblastoma with the emergence and progression of microarray technology and sequencing technology. The IDH1 mutant was found in an integrated genomic analysis in 2008 (Parsons et al., 2008). Many studies have been performed in recent years and suggest that mutated IDH1 participates in the pathogenesis of glioma. According to the WHO categorization of central nervous system tumors, glioblastoma is divided into IDH-mutant and IDH-wildtype subtypes (Louis et al., 2016). This categorization is based entirely on histological features. There are many specific genetic changes in glioblastoma cases, and the most frequently mutated or deregulated gene is epidermal growth factor receptor (EGFR), which is amplified in approximately 60% of glioblastomas (Huang et al., 2007). Many deregulations with certain pathways, such as PI3K, P53, and RB, have also been identified. Overall, these studies show the prospect of the gene signature in tumor diagnosis and prognosis, and they provide new evidence for tumor biology. With the progression of bioinformatics and open access of high-throughput data, researchers have studied multiple gene prognostic signatures for GBM, which result in more accuracy than single gene prognostic signatures (Colman et al., 2010; Yin et al., 2019).

The CNS has been considered as an immune-favored system based on the initial experimental data documented more than 50 years ago (Medawar, 1948; Billingham et al., 1954), but many findings have suggested that the immune microenvironment provides sufficient opportunities to treat brain tumors with immunotherapy even though the brain is an immunologically distinct region (Schiffer et al., 2017). Scientists have great interest in utilizing immunotherapy to treat glioblastoma because it has shown considerable improvements in the management of numerous solid tumors, including melanoma, renal cell carcinoma, and NSCLC. There are many ongoing clinical trials for immunotherapy, but the results are not satisfactory. Thus, we need more knowledge about the GBM immune microenvironment.

Herein, we constructed a robust seven immune-linked gene biosignature for risk stratification in glioblastoma patients. In contrast to a previous studies (Liang et al., 2020), we used univariate Cox regression analysis and LASSO regression assessment to classify genes as independent prognostic indicators. Among them, CTSB, NFKBIZ, TNFSF14, CXCL2, SEMA4F, and OSMR were characterized as high-risk genes, whereas BMPR1A was identified as low-risk gene.

The protease cathepsin B (CTSB) has been identified to highly express in cancer (Mijanovic et al., 2019), and associate with poor prognosis of a variety of cancers, including breast cancer, pancreatic cancer, and lung squamous cell carcinoma, which could be used as an independent predictor of these tumors (Gong et al., 2013; Zhang et al., 2014). It was previously found that the absence of CTSB delays the growth and invasion of pancreatic neuroendocrine tumors (Gocheva et al., 2006). Here, we identified CTSB as a risk pattern based on our risk model, which is in consistent with previous studies. NFKBIZ mutation is associated with ulcerative colitis, and the repeated inflammation and repair are closely related to the occurrence of colorectal cancer (Kakiuchi et al., 2020). Thus, chronic inflammation might be related to GBM. TNFSF14 is also known as LIGHT, which has been studied at preclinical level for more than 10 years and has shown the prospect of strengthening cancer immunotherapy (Skeate et al., 2020). CXCL2 can promote the recruitment of MDSC and is associated with the prognosis of bladder cancer (Zhang et al., 2017). SEMA4F is expressed in adults and related to the neural guidance of embryos. It can induce neurogenesis in prostate cancer, thus promoting cancer growth and migration (Ding et al., 2013). The cytokine receptor for oncostatin M (OSMR) regulates self-renewing brain tumor stem cells and promotes the resistance of GBM to ionizing radiation (Sharanek et al., 2020). In breast cancer, BMPR1-knockdown can inhibit RANKL production through p38 pathway, thereby inhibiting breast cancer-induced osteolysis (Liu Y. et al., 2018). Above all, the above mentioned seven genes play important roles in the occurrence and development of tumors.

We next created a landscape of 22 subtypes of immune cells and acquired the status of immune infiltration in the GBM microenvironment. Our results were similar to those of previous studies (Lu et al., 2019; Liang et al., 2020). Furthermore, we analyzed the relationship between the expression levels of seven immune-linked genes and the invading levels of six immune cells. The data demonstrated that the expression of these seven genes exhibited positive correlation with immune cell invasion (Supplementary Figure 4). All these findings indicated that our prognostic model may aid in understanding the immune status of glioblastoma patients. We also generated a circo plot to show the relationship between the risk score and expression levels of core immune checkpoints in GBM. This study may provide new targets or effective biomarkers for glioblastoma immunotherapy.

In summary, the immunotherapy of GBM patients should be individualized to obtain a better curative effect. Our study provides a prognosis prediction based on IRGs, which may reflect the immune status of GBM patients. However, our study had limitations as our study was based on databases and bioinformatics analyses. Immunohistochemistry, flow cytometry, and RT-PCR should be used to verify our research results.

## Conclusion

In our study, IRGs were identified to generate a prediction model of glioblastoma patient prognosis. We also explored the connection between these genes and the immune cells and immune checkpoints. Further research on these genes may provide new insights in GBM biology and promote immunotherapy.

## Data Availability Statement

The original contributions presented in the study are included in the article/Supplementary Material, further inquiries can be directed to the corresponding author/s.

## Author Contributions

LH and ZH performed all experiments, prepared figures, and drafted the manuscript. LH, ZH, XC, SW, and YF participated in data analysis and interpretation of results. LH and ZL designed the study and participated in data analysis. All authors have read and approved the manuscript.

## Conflict of Interest

The authors declare that the research was conducted in the absence of any commercial or financial relationships that could be construed as a potential conflict of interest.

## Footnotes

1 https://tcga-data.nci.nih.gov/tcga/
