# Supplementary material for: Expression Profile Analysis Identifies a Novel Seven Immune-Related Gene Signature to Improve Prognosis Prediction of Glioblastoma
Source: Front Genet. 2021 Feb 23;12:638458. doi: 10.3389/fgene.2021.638458 (PMC7940837; doi:10.3389/fgene.2021.638458)
Supplement: Supplementary file 1 [file Data_Sheet_1.PDF]

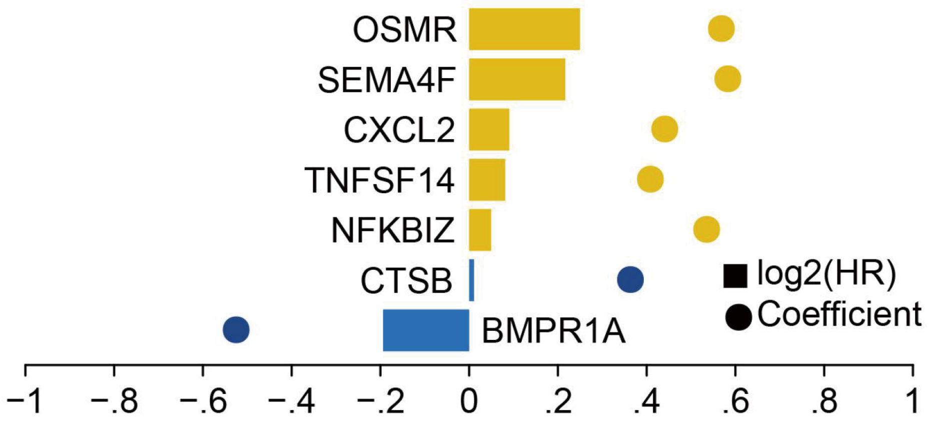

Figure S1. Establishment of prognostic gene signature by the univariate Cox regression and LASSO regression analysis. 7 prognostic IRGs screened out by univariate Cox regression and LASSO regression analysis.
