# Supplementary material for: Expression Profile Analysis Identifies a Novel Seven Immune-Related Gene Signature to Improve Prognosis Prediction of Glioblastoma
Source: Front Genet. 2021 Feb 23;12:638458. doi: 10.3389/fgene.2021.638458 (PMC7940837; doi:10.3389/fgene.2021.638458)
Supplement: Supplementary file 2 [file Data_Sheet_2.PDF]

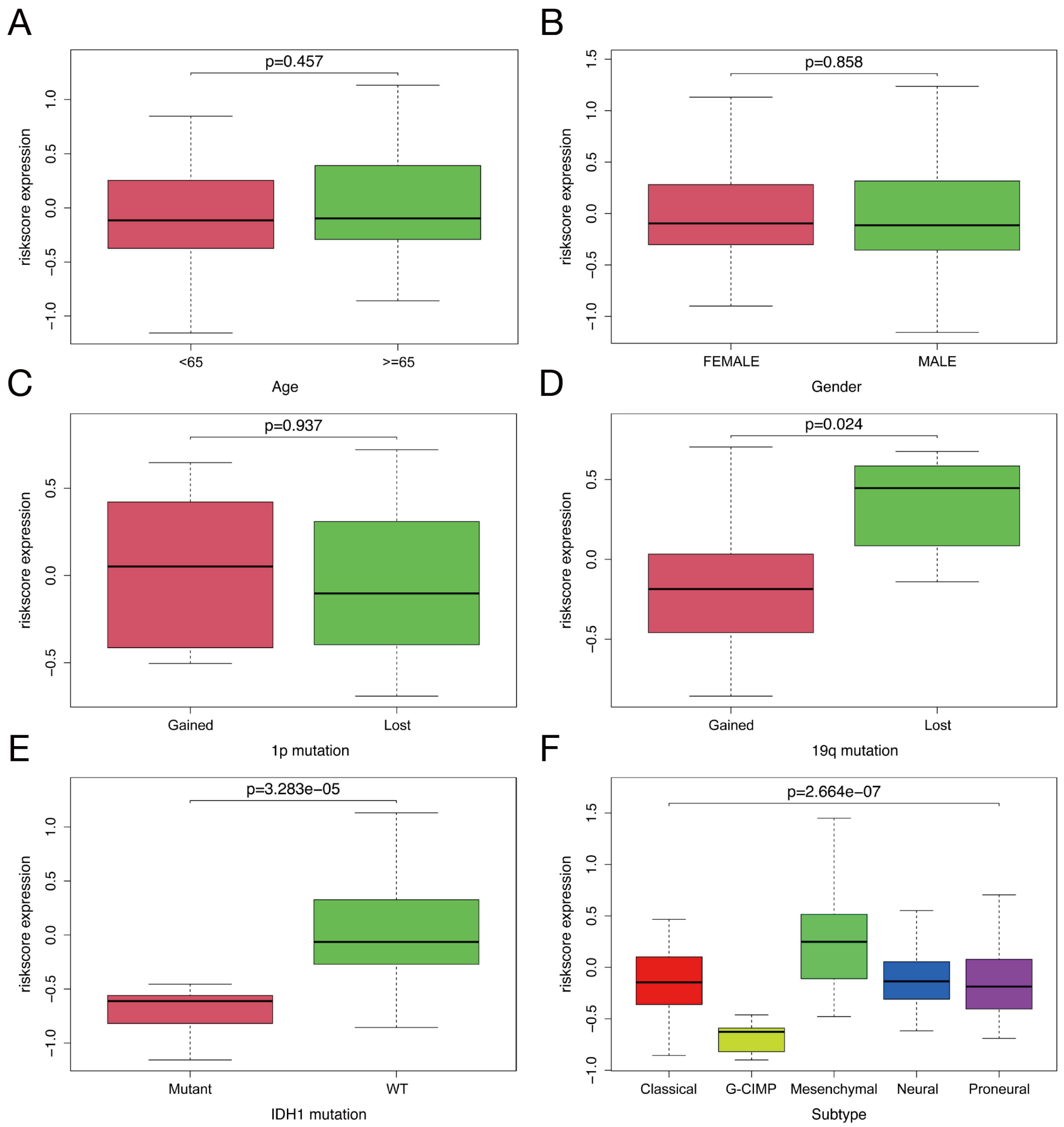

Figure S2. Relationship between the 7 IRG signature and clinical factors. The risk score had nothing to do with (A) Age, (B) Gender, and (C) 1p mutation. A higher risk score was associated with (D) 19q mutation, (E) IDH1 mutation, and (F) Subtype.
