# Supplementary material for: Expression Profile Analysis Identifies a Novel Seven Immune-Related Gene Signature to Improve Prognosis Prediction of Glioblastoma
Source: Front Genet. 2021 Feb 23;12:638458. doi: 10.3389/fgene.2021.638458 (PMC7940837; doi:10.3389/fgene.2021.638458)
Supplement: Supplementary file 3 [file Data_Sheet_3.PDF]

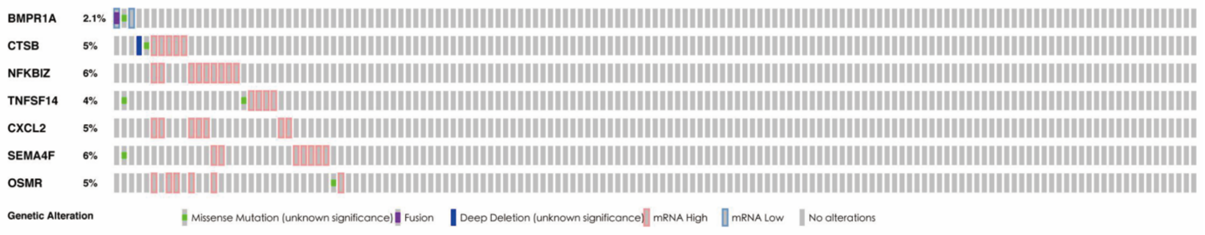

Figure S3. The genetic alterations of seven-immune-related genes in the TCGA cohort of GBM. NFKBIZ and SEMA4F are the genes with the highest mutation frequency. And there were 5 genes with a mutation rate ≥ 5%.
