# Supplementary material for: Expression Profile Analysis Identifies a Novel Seven Immune-Related Gene Signature to Improve Prognosis Prediction of Glioblastoma
Source: Front Genet. 2021 Feb 23;12:638458. doi: 10.3389/fgene.2021.638458 (PMC7940837; doi:10.3389/fgene.2021.638458)
Supplement: Supplementary file 4 [file Data_Sheet_4.PDF]

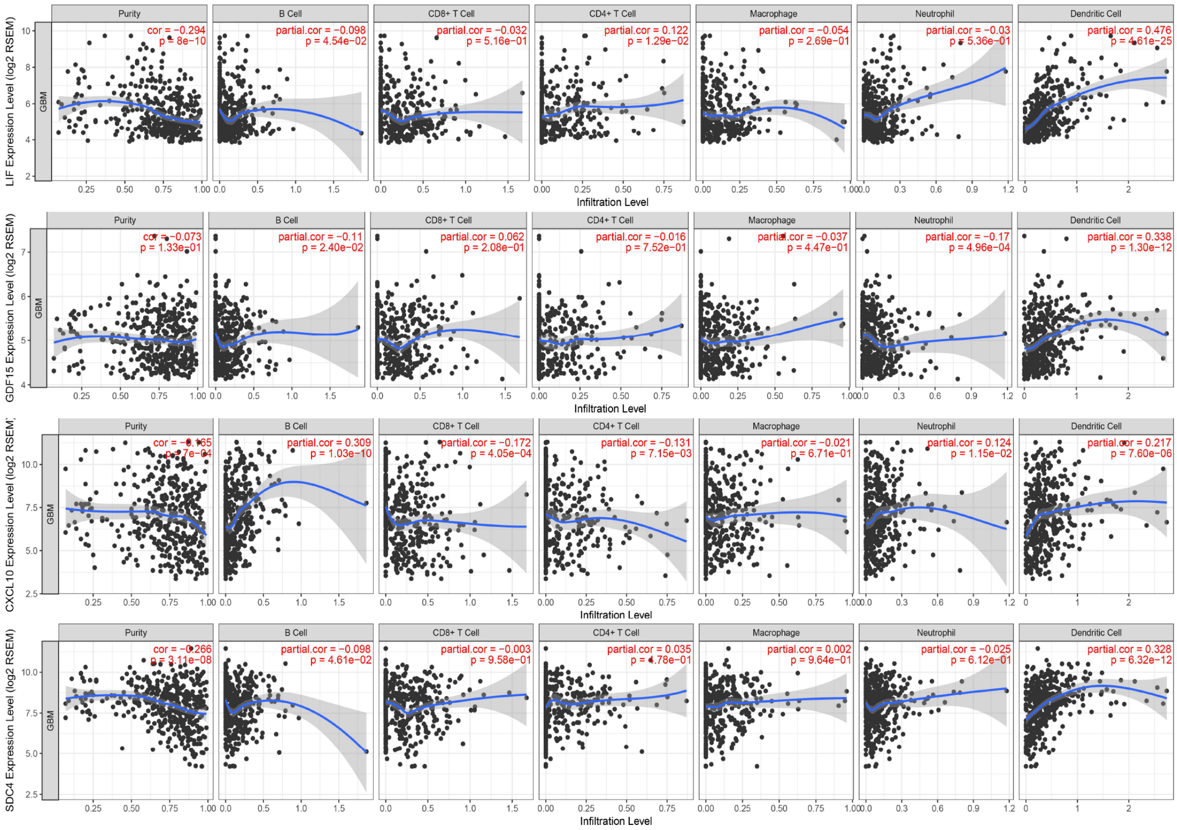

Figure S4. Correlations of seven-immune-related gene expression with immune infiltration level in GBM (TIMER). The scatter plots identify the different profiles of immune cells associated with seven-immune-related gene. These seven-immune-related gene expression is significantly related to tumor purity and has significant positive correlations with immune cell infiltration.
