# Supplementary material for: Expression Profile Analysis Identifies a Novel Seven Immune-Related Gene Signature to Improve Prognosis Prediction of Glioblastoma
Source: Front Genet. 2021 Feb 23;12:638458. doi: 10.3389/fgene.2021.638458 (PMC7940837; doi:10.3389/fgene.2021.638458)
Supplement: Supplementary file 5 [file Data_Sheet_5.pdf]

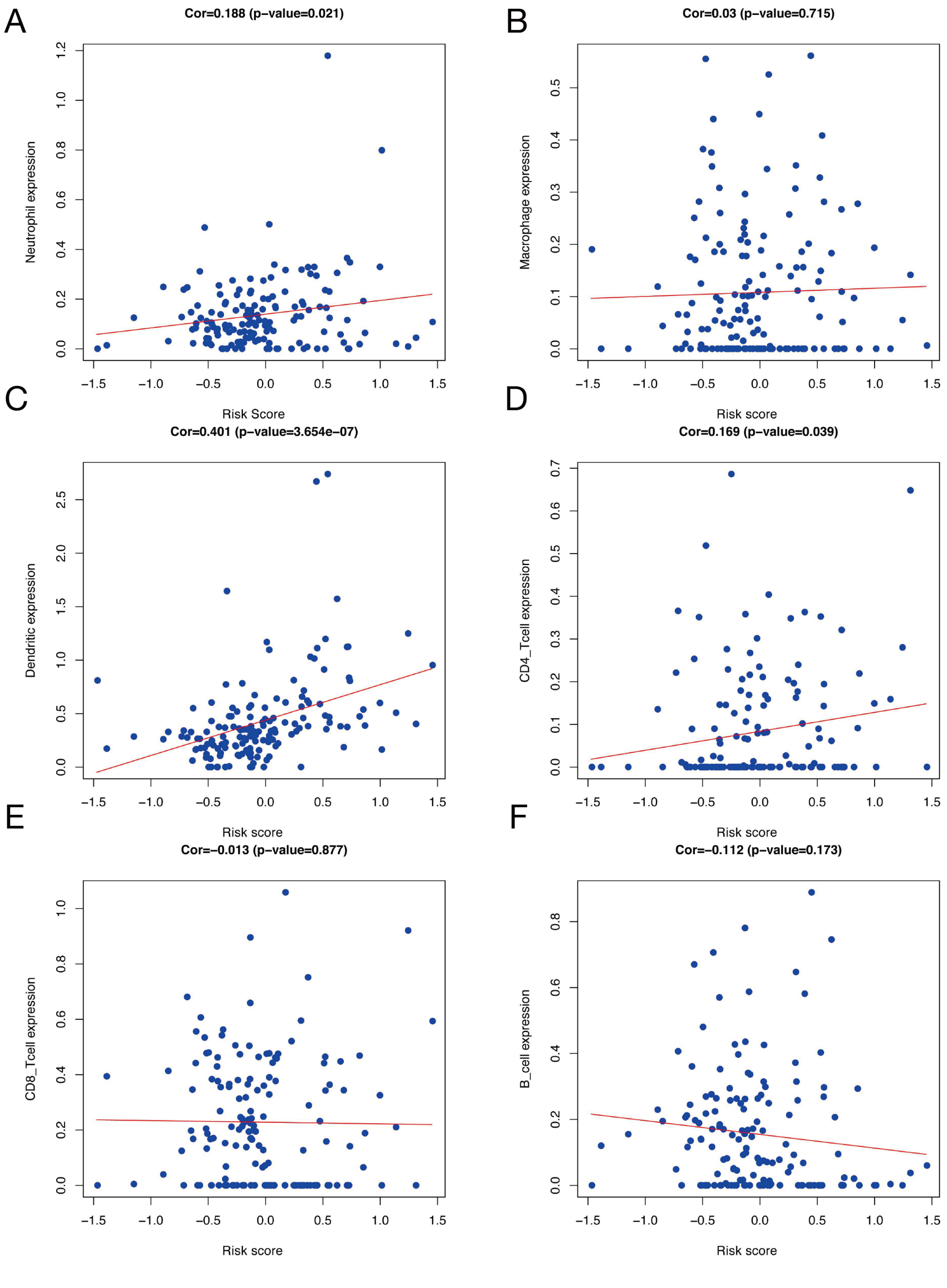

Figure S5. The association between the IRGs signature and tumor-infiltrating immune cells. (A) Neutrophil cells. (B) Macrophage. (C) Dendritic cells. (D) CD4+ T cells. (E) CD8+ T cells. (F) B cells.
