# Supplementary material for: Expression Profile Analysis Identifies a Novel Seven Immune-Related Gene Signature to Improve Prognosis Prediction of Glioblastoma
Source: Front Genet. 2021 Feb 23;12:638458. doi: 10.3389/fgene.2021.638458 (PMC7940837; doi:10.3389/fgene.2021.638458)
Supplement: Supplementary file 6 [file Data_Sheet_6.pdf]

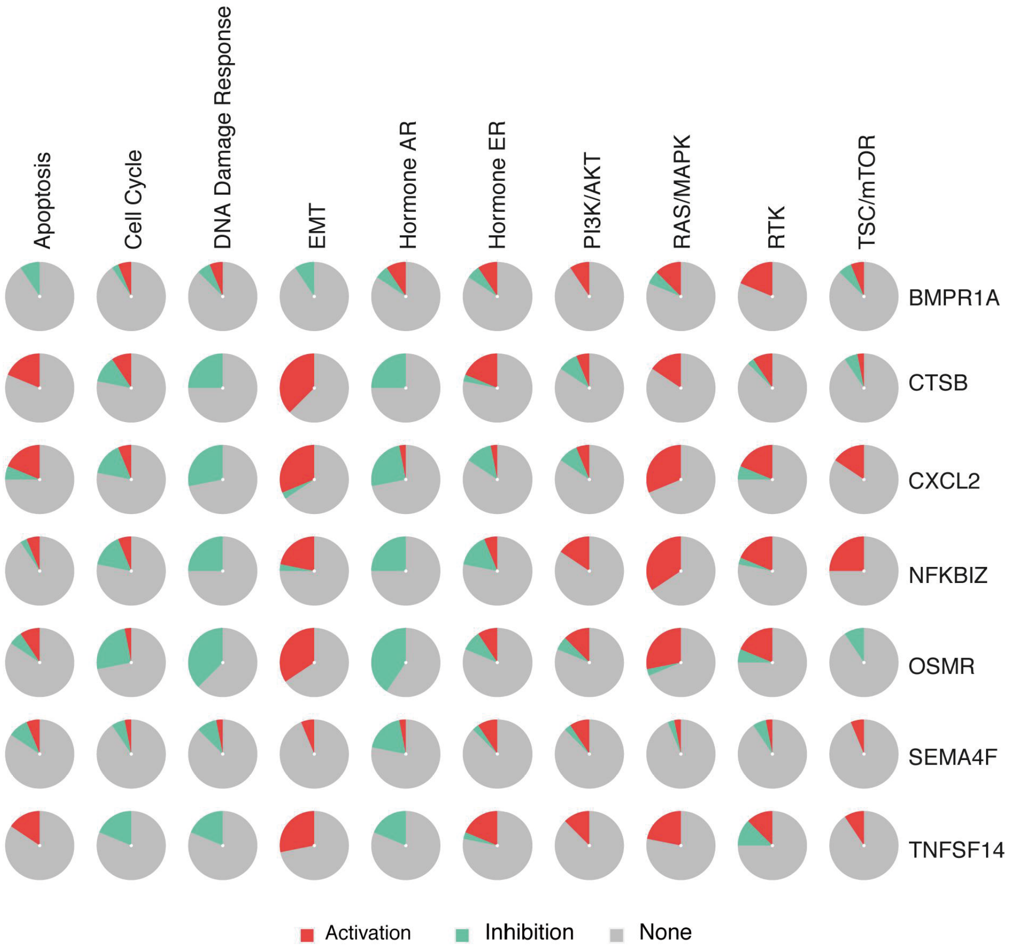

Figure S6. Correlations between the seven individual genes and ten key signaling pathways in GBM (GSCALite). Seven genes are highly associated with activation or inhibition of multiple oncogenic pathways, mainly associated with the activity of apoptosis, cell cycle, DNA damage response, EMT, Hormone ER, and RAS/MAPK pathways.
