# Supplementary material for: Expression Profile Analysis Identifies a Novel Seven Immune-Related Gene Signature to Improve Prognosis Prediction of Glioblastoma
Source: Front Genet. 2021 Feb 23;12:638458. doi: 10.3389/fgene.2021.638458 (PMC7940837; doi:10.3389/fgene.2021.638458)
Supplement: Supplementary file 7 [file Data_Sheet_7.PDF]

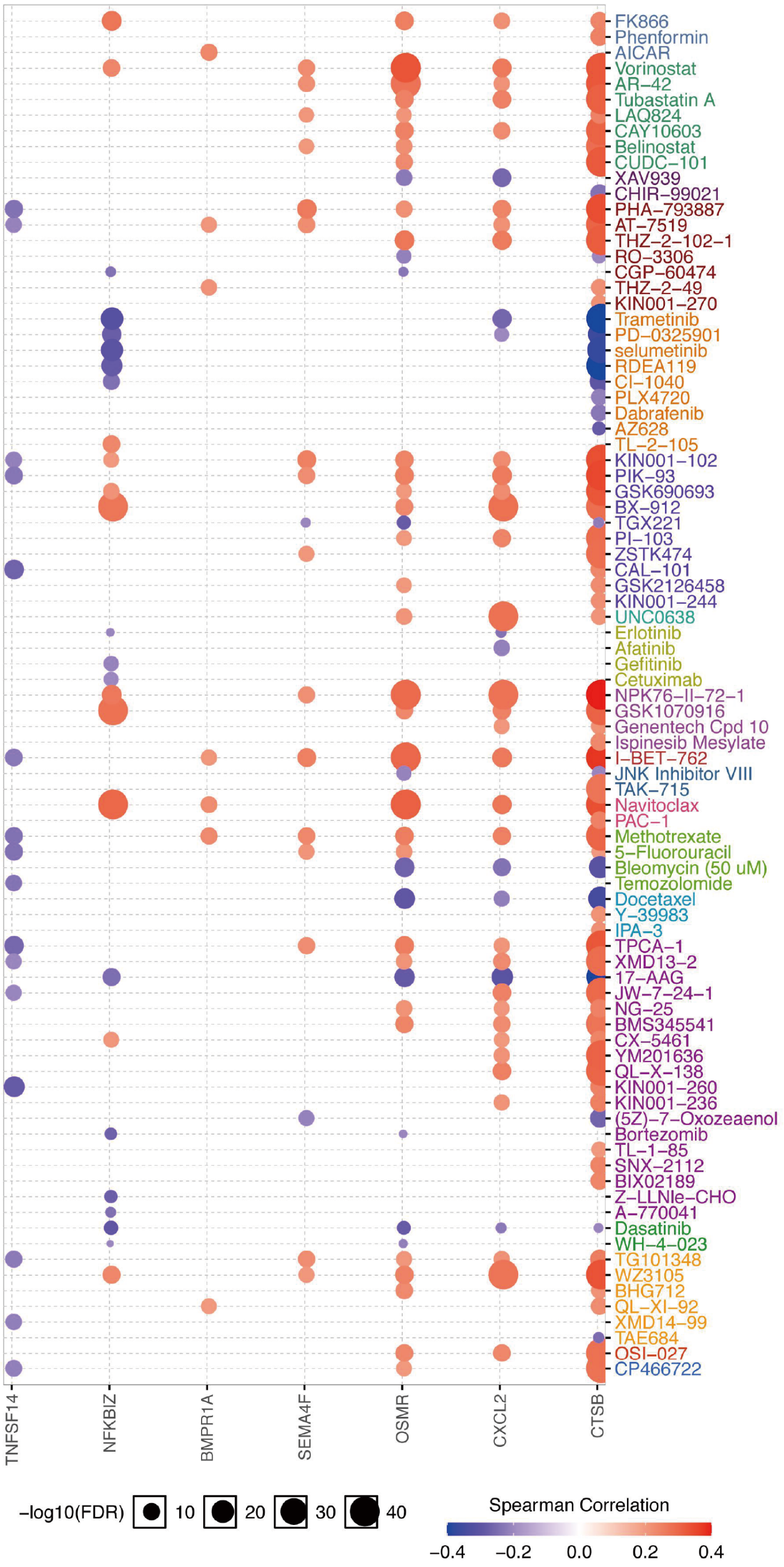

Figure S7. The drug resistance analysis of seven-immune-related genes based on GDSC IC50 drug data (GSCALite). The Spearman correlation represent the gene expression correlates with the drugs. The red dots represent sensitivity to the drugs, while blue dots represent the opposite.
